# Cooling Capacity Test for MIL-101(Cr)/CaCl_2_ for Adsorption Refrigeration System

**DOI:** 10.3390/molecules25173975

**Published:** 2020-08-31

**Authors:** Zhongbao Liu, Banghua Zhao, Yong Huang, Xin Qi, Fengfei Lou

**Affiliations:** 1 Department of Refrigeration and Cryogenic Engineering, College of Environmental and Energy Engineering, Beijing University of Technology, 100 Pingleyuan Road, Chaoyang, Beijing 100124, China; zhaobanghua@emails.bjut.edu.cn (B.Z.); loufengfei@emails.bjut.edu.cn (F.L.); 2 Suzhou Sujing Anfa Air Conditioning Co. LTD, 2 Weixin Road, Suzhou Industrial Park, Suzhou 215112, China; HY@aimfar.com.cn; 3 China Household Electric Appliance Research Institute, 6 Yuetan beixiao Str, Xicheng, Beijing 100037, China; qix@cheari.com

**Keywords:** MIL-101(Cr)/CaCl_2_-20%, composite materials, modeling and simulation, adsorption performance

## Abstract

An MIL-101(Cr) powder material was successfully prepared using the hydrothermal synthesis method, and then the original MIL-101(Cr) was combined with different mass fractions of CaCl_2_ using the immersion method to obtain a MIL-101(Cr)/CaCl_2_ composite material. The physical properties of the adsorbent were determined by X-ray powder diffraction (XRD), an N2 adsorption desorption isotherm test, and thermogravimetric analysis (TG). The water vapor adsorption performance of the metal-organic frameworks MOFs was tested with a gravimetric water vapor adsorption instrument to analyze its water vapor adsorption mechanism. Based on the SIMULINK platform in the MATLAB software, a simulation model of the coefficient of performance (COP) and cooling capacity of the adsorption refrigeration system was established, and the variation trends of the COP and cooling capacity of the adsorption refrigeration system under different evaporation/condensation/adsorption/desorption temperatures was theoretically studied. MIL101-(Cr)/CaCl_2_-20% was selected as the adsorption material in the adsorption refrigeration system through the physical characterization of composite materials with different CaCl_2_ concentrations by means of adsorption water vapor test experiments. A closed adsorption system performance test device was built based on the liquid level method. The cooling power per unit and adsorbent mass (COP and SCP) of the system were tested at different evaporation temperatures (288 K/293 K/298 K); the adsorption temperature was 298 K, the condensation temperature was 308 K, and the desorption temperature was 353 K. The experimental results showed that COP and SCP increased with the increase in the evaporation temperature. When the evaporation temperature was 298 K, the level of COP was 0.172, and the level of SCP was 136.9 W/kg. The COP and SCP of the system were tested at different adsorption temperatures (293 K/298 K/303 K); the evaporation temperature was 288 K, the condensation temperature was 308 K, and the desorption temperature was 353 K. The experimental results showed that the levels of COP and SCP decreased with the increase in the adsorption temperature. When the adsorption temperature was 293 K, the level of COP was 0.18, and the level of SCP was 142.4 W/kg.

## 1. Introduction

Adsorption refrigeration is an environmentally friendly refrigeration method. Adsorption refrigerators can use natural working fluids as refrigerants and have zero Ozone Depletion Potential (ODP), which has attracted widespread attention in recent years. Low-temperature heat-driven environmentally friendly adsorption refrigeration systems are becoming a viable alternative to electric-driven vapor compression refrigeration systems. The low cooling capacity of the technology hinders its successful commercialization [1,2,3].

At present, there are two main ways to improve the performance of an adsorption refrigeration system. One is to optimize the circulation of the system, and the other is to improve the adsorption performance of the working medium [4]. Metal-organic frameworks (MOFs) are a new type of solid adsorbent with a high specific surface area (4000 m^2^·g^−1^) and porosity, which have broad application prospects in TDCs or adsorption heat pumps (AHPs) [5]. The following is a review of the literature on adsorption refrigeration studies using metal-organic frameworks (MOFs) as adsorbents. Xia [6] synthesized three Zr-MOFs with different pore sizes: UiO-66, UiO-67 and NU-1000, and determined their adsorption isotherms to water and ethanol. The cooling power per unit (COP) of MOF/water and MOF/ethanol were studied by adsorption system model. The results showed that the COP of MOF/water was generally higher than that of MOF/ethanol, but the performance of MOF/ethanol was better than that of MOF/water. Ehrenman [7] tested the water absorption capacity and cyclic stability of the high-porosity metallic organic framework material MIL-101 (3D-[Cr_3_F(H_2_O)_2_O(bdc)_3_∙25H_2_O]) at 25 °C. An N_2_ adsorption isotherm showed that, after 20 adsorption and desorption cycles, the specific surface area of the Brunauer-Emmett-Teller (BET) of MIL-101(Cr) decreased from 2059 m^2^/g to 2047 m^2^/g, and its water absorption capacity decreased by 1.9%. Ahmed Rezk [8] studied the water absorption properties of six different MOF materials (Cu-BTC, Fe-BTC, MIL-53, Birm-1, Birm-1-K and Birm-1-Li). The results show that the surface areas of Cu-BTC and Fe-BTC materials are 2100 m^2^/g and 1600 m^2^/g, respectively, which have the highest water absorption performance. Ahmed Rezk [9] also reported the isothermal adsorption properties of six different MOF materials (MIL-101(Cr), MIL-100 (Cr), MIL-53 (Cr), CPO-27-Ni, Cu-BTC, Fe-BTC) and silica gel to ethanol. The adsorption isotherms of various adsorbents were measured at 25 °C; the results showed that MIL-101(Cr) was superior to other adsorbents in ethanol adsorption. The stability of MIL-101(Cr)/ethanol in 20 consecutive adsorption–desorption cycles was also studied; the results show that the working pair has good stability. Baosheng Shi [10] compared the adsorption properties of silica gels RD-2060, SAPO-34 and CPO-27 (Ni). Under the condition of 0.1 relative humidity, the water vapor absorption rate of CPO-27 (Ni) was 0.41 kg/kg, 86.3% higher than SAPO-34, and 7 times higher than RD-2060. Teo [11] modified MIL-101(Cr) with 5%, 20% and 40% alkali metal ions (Li^+^, Na^+^, K^+^), respectively, to test the properties of MOF materials after modification. Garzon-tovar [12] synthesized a new solid composite adsorbent by combining metal organic framework UiO-66 and hygroscopic salt CaCl_2_ through spray drying, improving the water absorption capacity of the original material. At the same time, composite adsorption and materials were applied in an adsorption refrigeration system, and the results showed that the SCP was 631 W/kg and the COP was 0.83. Jian Yan [13] developed a new composite material (MIL-101@GO) for adsorption heat pump (AHPs). The steam adsorption isotherm and adsorption kinetics of the composite materials were determined by the weight method. The results showed that MIL-101@GO has a super high water vapor adsorption capacity, and its adsorption capacity can reach 1.58 g/g. Six consecutive adsorption cycle experiments showed that the synthesized MIL-101@GO has good reversibility and stability for water vapor adsorption.

Calcium chloride is a very hygroscopic salt, it has a strong affinity for water. It has been reported in the literature that when salt is combined with activated carbon, silica gel, zeolite and other porous materials (matrix) to form a composite material, its performance is significantly improved. Aristov [14] discusses a silica gel/CaCl_2_ composite material called a selective water absorber (SWS-1L) in which the enhanced water vapor capacity increases in COP from 0.48 to 0.7 at the desorption temperature of 90 °C, evaporation temperature of 5 °C, and condensation temperature and adsorption temperature of 40 °C. Wang [15] combined activated carbon fiber felts (ACF FELT) with 30% calcium chloride, and the water adsorption capacity reached 1.7 g^H2O^/g^ads^, higher than activated carbon, silica gel and even the compound of silica gel and calcium chloride. Chan [16] soaked zeolite 13X in 46% CaCl_2_ solution, and the adsorption capacity increased by 0.4 g^H2O^/g^ads^ at 25 and 75 °C and 870 Pa.

In this paper, MIL-101(Cr)/CaCl_2_ composite materials with different CaCl_2_ concentrations (10%, 20%, 30%) were prepared by the dipping method. Through physical characterization and water vapor adsorption performance experiments, MIL-101(Cr)/CaCl_2_-20%/water was selected as the pair of working media for the adsorption refrigeration system to test the system performance at different evaporation/adsorption temperatures.

## 2. Results

### 2.1. XRD Analysis

**Figure 1:**
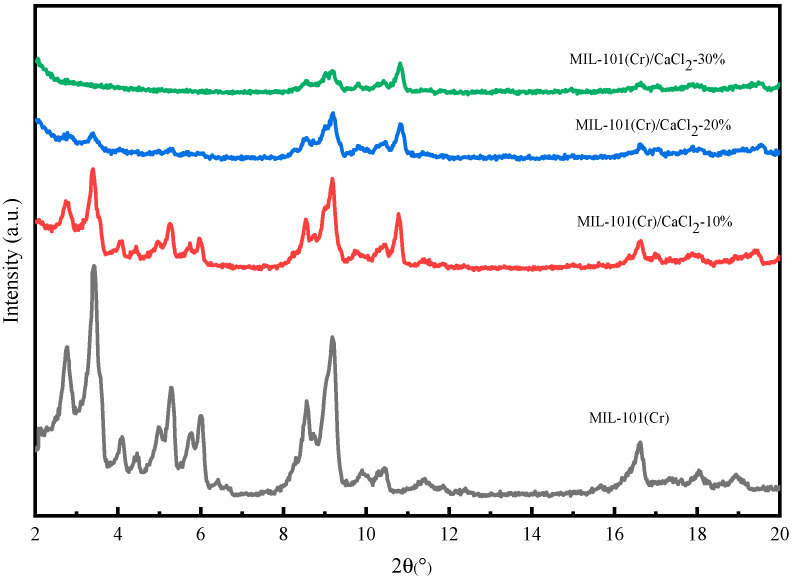
XRD pattern of MIL-101(Cr) and composites.

Figure 1 shows the XRD spectra of MIL-101(Cr)/CaCl_2_ composites. It can be seen from the figure that the main characteristic diffraction peaks of MIL-101(Cr) samples appear at 2θ = 3.42°, 5.98°, 8.56°, 9.18°, 10.30° and 16.54°, and the position of the diffraction peak is very consistent with the report [15]. The MIL-101(Cr)/CaCl_2_ composite has the same diffraction peak as the original MIL-101(Cr), indicating that MIL-101(Cr) is still the main component of the MIL-101(Cr)/CaCl_2_ composite. In addition, we can also find that the peak of XRD diffraction gradually decreases with the increase in CaCl_2_ content, because the disordered hydrated salt molecules are filled into the mesopores of MIL-101(Cr) with the increase in CaCl_2_ content [17].

### 2.2. TG Analysis

**Figure 2:**
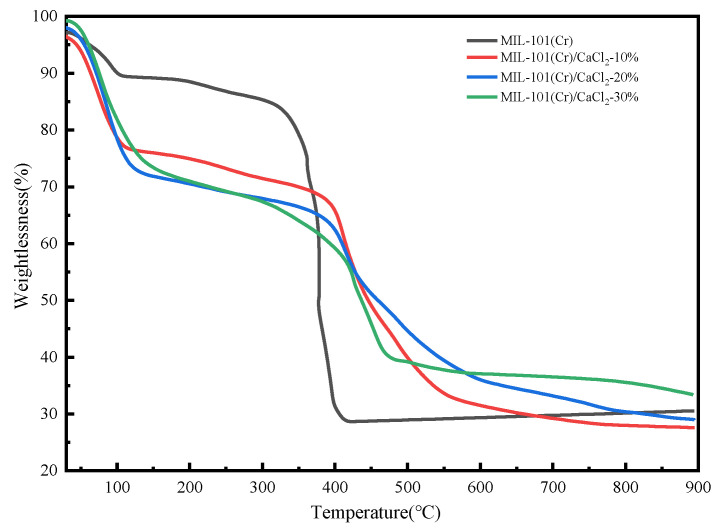
Thermogravimetric TG curve of MIL-101(Cr) and composites.

The thermogravimetric results for MIL-101(Cr)/CaCl_2_ composites are shown in Figure 2. The weightlessness of the material mainly goes through two stages. As the temperature rises to 100 °C, the water in MIL-101(Cr) dissipates, and the weight loss in the first stage is caused by the loss of calcium chloride in the composite. At 300 °C, MIL-101(Cr) began to lose the crystal water in the structure, and by 400 °C, the framework was decomposed. The structure of the composite material was destroyed at 450 °C, which was delayed by 50 °C compared with MIL-101(Cr).

### 2.3. N_2_ Adsorption–Desorption Isotherms and Pore Size Analysis

**Figure 3:**
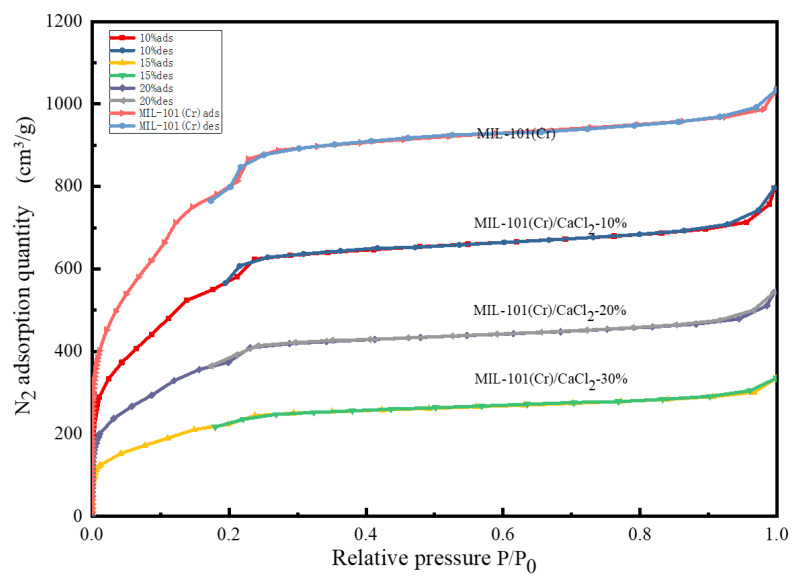
Nitrogen adsorption–desorption isotherms of MIL-101(Cr) and composites.

Figure 3 shows the N_2_ adsorption and desorption isotherms for the MIL-101(Cr)/CaCl_2_ composites. According to the classification of the BET adsorption isotherm curve and type-II adsorption–desorption isotherms, in the low pressure stage (*p* = 0–0.2/*p*_0_), the nitrogen adsorption quantity displays sharp growth, proving that it contains a certain amount of microporous materials. At a relative pressure of about 0.2, the material adsorbed a second time, indicating that the experimentally synthesized material contained two different cage structures [18]. The adsorption capacity in the middle section of the curve (*p*/*p*_0_ = 0.2–0.9) increased slowly with the increase in pressure. In the second half of the curve (*p*/*p*_0_ = 0.9–1.0), the adsorption line rose sharply and did not show adsorption saturation until the vapor pressure was close to saturation, indicating that the sample contained a certain amount of mesoporous and macroporous bulk filling due to capillary condensation.

**Figure 4:**
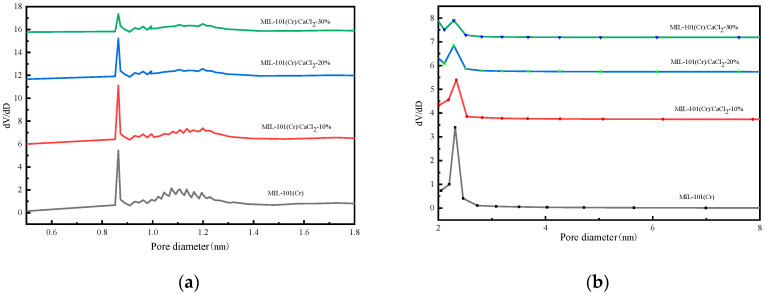
Pore diameter distribution of MIL-101(Cr) and MIL-101(Cr)/CaCl_2_ composites: (**a**) micropore, (**b**) mesoporous.

Figure 4 shows the pore size distribution of MIL-101(Cr) and MIL-101(Cr)/CaCl_2_ composites. It can be seen from the figure that the MIL-101(Cr)/CaCl_2_ composite pore diameters are mainly distributed at 0.86 nm, 1.19 nm and 2.32 nm. Table 1 lists the pore structure parameters of the MIL-101(Cr) and MIL-101(Cr)/CaCl_2_ composites. With the increase in CaCl_2_ addition, the specific surface area, total pore volume and pore size distribution of microporous mesopores of MIL-101(Cr)/CaCl_2_ composites decreased gradually. This is because the added CaCl_2_ molecules occupy part of the pores of the MIL-101(Cr) material. For MIL-101(Cr)/CaCl_2_-30%, the BET specific surface area and total pore volume were only 193 m^2^/g and 0.071 cm^3^/g, which indicates that the CaCl_2_ molecule almost completely blocked the pores of MIL-101(Cr).

### 2.4. Water Vapor Adsorption Isotherms

**Figure 5:**
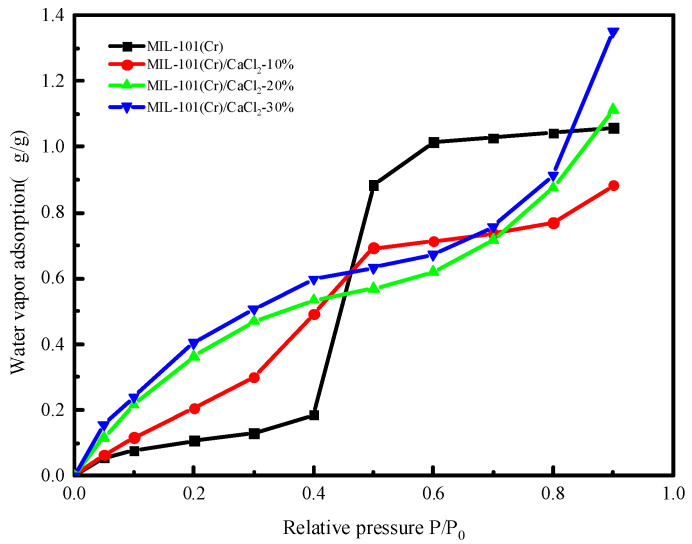
Water sorption isotherms of MIL-101(Cr) andMIL-101(Cr)/CaCl_2_ composites at 298 K.

It can be seen from Figure 5 that the MIL-101(Cr)/CaCl_2_-10% adsorption isotherm line type is almost the same as the MIL-101(Cr) line type. The adsorption isotherms of MIL-101(Cr)/CaCl_2_-20% and MIL-101(Cr)/CaCl_2_-30% showed obvious changes, and the equilibrium adsorption capacity was higher than that of the original MIL-101(Cr). As can be seen from the adsorption isotherm, water absorbing capacity of calcium chloride itself leads to the low pressure area which in the water vapor adsorption experiment showed higher adsorption performance, the relative pressure adsorption amount compared with the original start going down after 0.4. This is due to the good water absorption characteristics of calcium chloride. The specific surface area of the material gradually became dominant; the specific surface area of the composites compared with MIL-101 decreased, so the relative pressure was greater than 0.4 after the adsorption performance was reduced. In particular, the MIL-101(Cr)/CaCl_2_-20% and MIL-101(Cr)/CaCl_2_-30% composite materials had a higher water adsorption capacity and equilibrium adsorption capacity than the original MIL-101(Cr) at low pressure. Therefore, the MIL-101(Cr)/CaCl_2_-20% and MIL-101(Cr)/CaCl_2_-30% materials are very attractive adsorbent materials for use in adsorption refrigeration applications. Although the equilibrium water adsorption capacity of the MIL-101(Cr)/CaCl_2_-30% composite adsorbent is high, it becomes agglomerated after water absorption continues to agglomerate after repeated drying, which significantly affects the material’s adsorption capacity and limits its use in adsorption refrigeration applications. In summary, the MIL-101(Cr)/CaCl_2_-20% composite material was selected for its adsorption refrigeration performance.

### 2.5. Analysis of the Simulation Results

#### 2.5.1. Effect of Different Desorption Temperatures on System Cooling Capacity and COP

**Figure 6:**
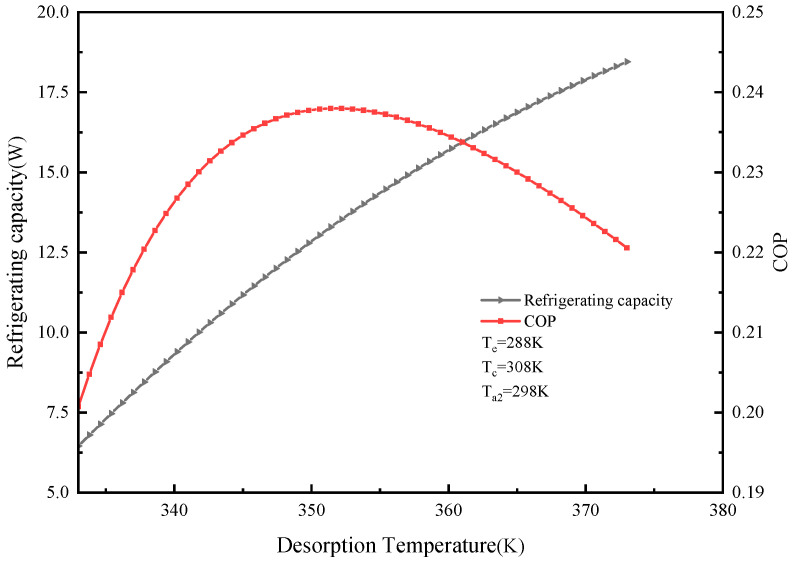
Effect of desorption temperature on refrigeration capacity and coefficient of performance (COP).

The effect of the desorption temperature T_g2_ on cooling capacity and COP is shown in Figure 6. It can be seen from the figure that the cooling capacity of the system increased with the increase in the desorption temperature. The total cycle time was 9000 s. From the COP, there was an optimal desorption temperature. When T_e_ = 288 K, T_c_ = 308 K, Ta_2_ = 298 K, the optimal desorption temperature was 351 K. At this time, the system cooling capacity was 118 kJ and the COP was 0.24. Before the optimum desorption temperature, the COP value increased with the desorption temperature in a certain temperature range. The higher the desorption temperature, the more water vapor is desorbed from the adsorption bed, so the more water vapor is adsorbed by the material. The more the corresponding cooling capacity, the greater the amount of cooling. However, when the desorption temperature exceeds the optimum desorption temperature, the COP decreases as the desorption temperature increases because the desorption temperature increases and the heat consumed by the system cycle also increases, causing the COP to decrease.

#### 2.5.2. Effect of Different Condensation Temperatures on System Cooling Capacity and COP

**Figure 7:**
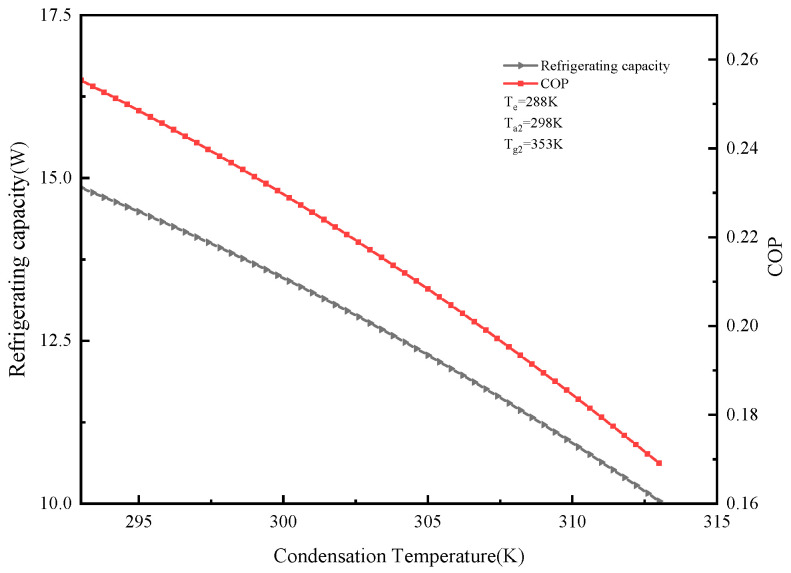
Effect of condensation temperature on refrigeration capacity and COP.

Calculation parameters: T_e_ = 288 K, T_a2_ = 298 K, T_g2_ = 353 K. The effect of condensation temperature Tc on cooling capacity and COP is shown in Figure 7. It can be seen from the figure that as the condensing temperature increases, the cooling capacity and COP of the system decrease. When T_c_ = 313 K, the cooling capacity was 10.04 W and the COP was 0.17. This is because the condensation temperature is not conducive to the condensation of the refrigerant vapor, reducing the desorption amount of the refrigerant, thereby reducing the COP of the system. At the same time, the adsorption delay occurs when the condensation temperature rises, so that the adsorption amount decreases, causing the cooling capacity to decrease accordingly. It can be seen from the above law that reducing the condensation temperature of the adsorption refrigeration system can improve the performance of the system.

#### 2.5.3. Effects of Different Adsorption Temperatures on Cooling Capacity and COP of the System

**Figure 8:**
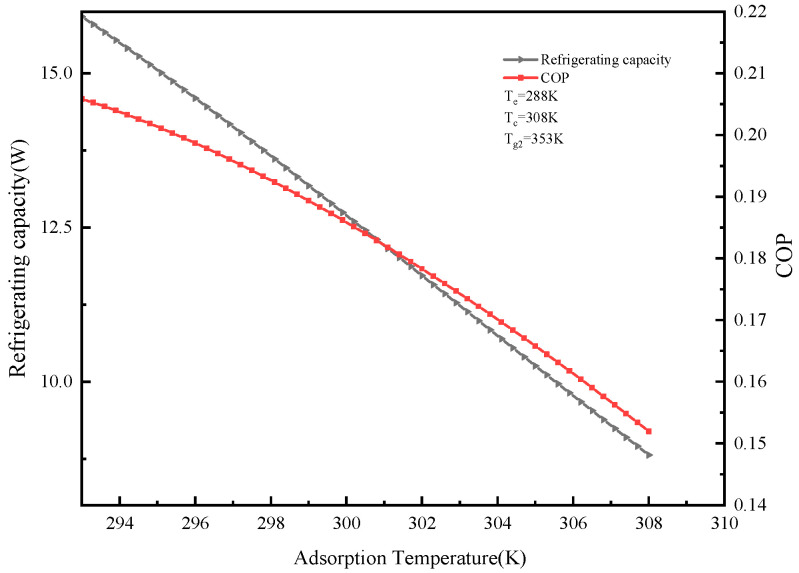
Effect of adsorption temperature on refrigeration capacity and COP.

Calculation parameters: T_e_ = 288 K, T_c_ = 308 K, T_g2_ = 353 K. The effect of condensation temperature T_a2_ on cooling capacity and COP is shown in Figure 8. It can be seen from the figure that as the adsorption temperature increases, the cooling capacity and COP of the system decrease. The adsorption temperature increases, so that the difference between the adsorption temperature and the initial adsorption temperature decreases, and then the cyclic adsorption amount also decreases, eventually leading to a decrease in system COP. When T_e_ = 288 K, T_c_ = 308 K, T_g2_ = 353 K, and T_a2_ = 308 K, the minimum cooling capacity and COP were 8.81 W and 0.15, respectively.

#### 2.5.4. Effects of Different Evaporation Temperatures on Cooling Capacity and COP of the System

**Figure 9:**
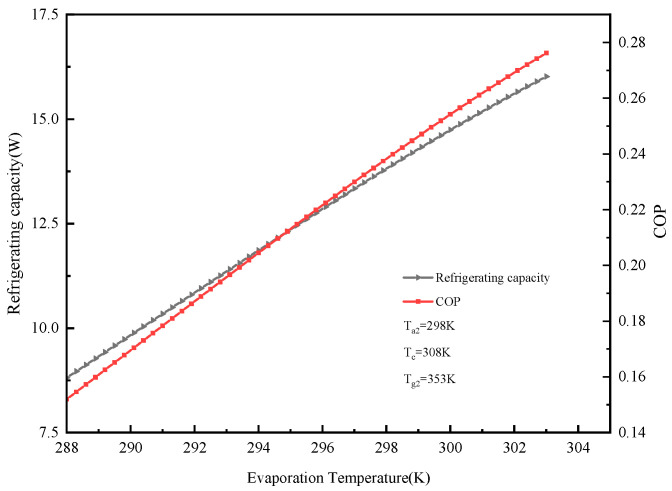
Effect of evaporation temperature on refrigeration capacity and COP.

Calculation parameters: T_a2_ = 298 K, T_c_ = 308 K, T_g2_ = 353 K. The effect of evaporation temperature (T_e_) on the cooling capacity and COP is shown in Figure 9. It can be seen from the figure that the system’s cooling capacity and coefficient of performance COP both increase with the increase in the evaporation temperature. When the evaporation temperature increased from 288 K to 303 K, the cooling capacity and COP—initially 8.81 W and 0.15—increased to 16 W and 0.28. This occurs because (1) the higher the evaporation temperature, the higher the evaporation pressure; (2) the larger the pressure difference in the adsorption bed, the smaller the mass transfer resistance; (3) the larger the adsorption amount of the adsorbent, the larger the sensible heat of the adsorption bed. Ultimately, the cooling capacity and coefficient of COP performance both increase as the evaporation temperature increases.

### 2.6. Analysis of the Experimental Results

#### 2.6.1. System Performance Analysis at Different Evaporation Temperatures

**Figure 10:**
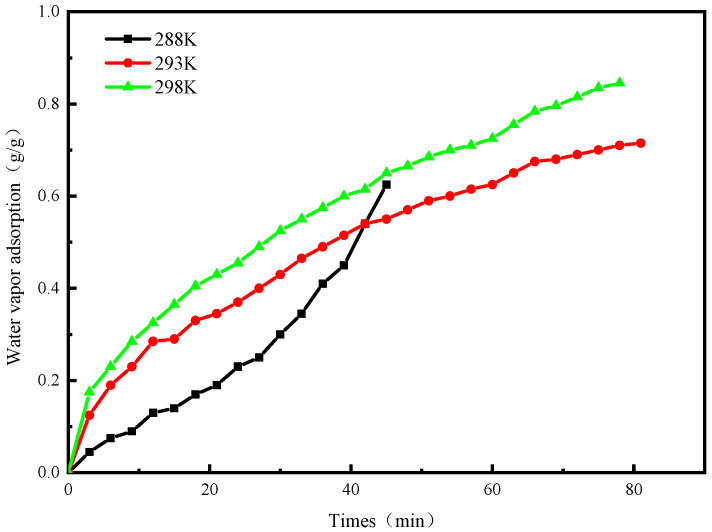
The curve of adsorption uptake of water vapor versus time.

Figure 10 shows the water vapor adsorption capacity of the adsorption refrigeration system over time at different evaporation temperatures. It can be seen from the figure that when T_a2_ = 298 K, T_c_ = 308 K, T_g2_ = 353 K, the evaporation temperature was 288 K, the adsorption capacity was 0.625 g/g; when the evaporation temperature was 293 K, the adsorption capacity was 0.715 g/g; when the evaporation temperature was 298 K, the adsorption capacity was 0.845 g/g. We can find that as the evaporation temperature increases, the equilibrium adsorption amount of water vapor temperature also increases. This is because the evaporation pressure increases and the pressure difference in the refrigeration system also increases, which is beneficial to MIL-101(Cr)/CaCl_2_-20% composite adsorption.

**Figure 11:**
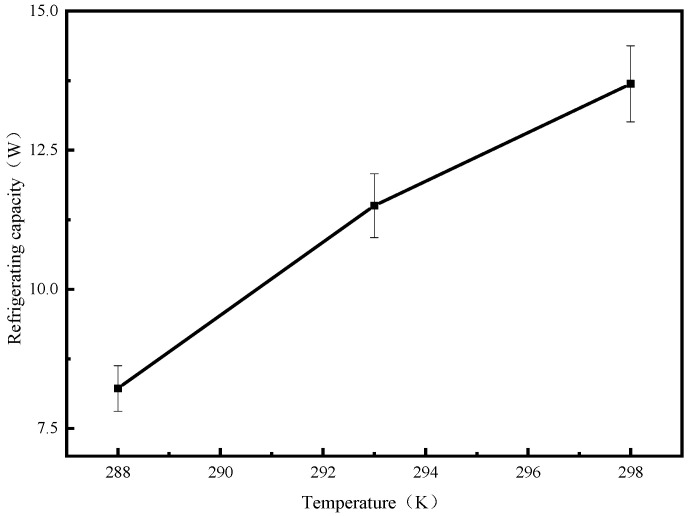
Cooling capacity of the system at different evaporation temperatures.

It can be seen from Figure 11 that as the evaporation temperature increases, the cooling capacity increases. When the evaporation temperature was 298 K, the cooling capacity of the system increased by 5.47 W compared with that of the system when the evaporation temperature was 288 K.

**Figure 12:**
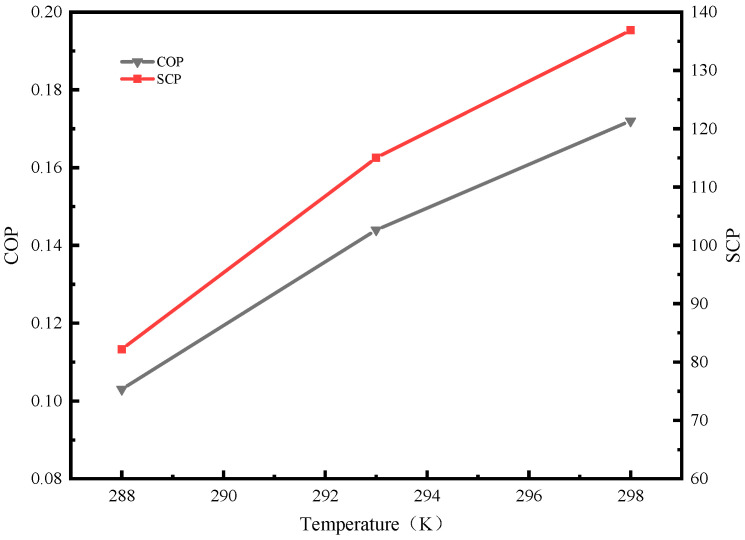
COP and adsorbent mass (SCP) of the system at different evaporation temperatures.

During the experiment, as the evaporation temperature increases, the system cooling capacity increases, resulting in an upward trend in the system’s COP and SCP. It can be found from Figure 12 that the COP of the system was 0.103 when the evaporation temperature was 288 K. When the evaporation temperature rose to 298 K, the COP of the system was 0.172, which was increased by 67%. For SCP, when the evaporation temperature was 288 K, the value was 82.2 W/kg, and when the evaporation temperature was 298 K, the SCP value was 136.9 W/kg, which is an increase of nearly 66.5%. Therefore, in the refrigeration process, in the case of meeting the cooling demand, the evaporation temperature of the system should be increased as much as possible.

#### 2.6.2. System Performance Analysis at Different Adsorption Temperatures

**Figure 13:**
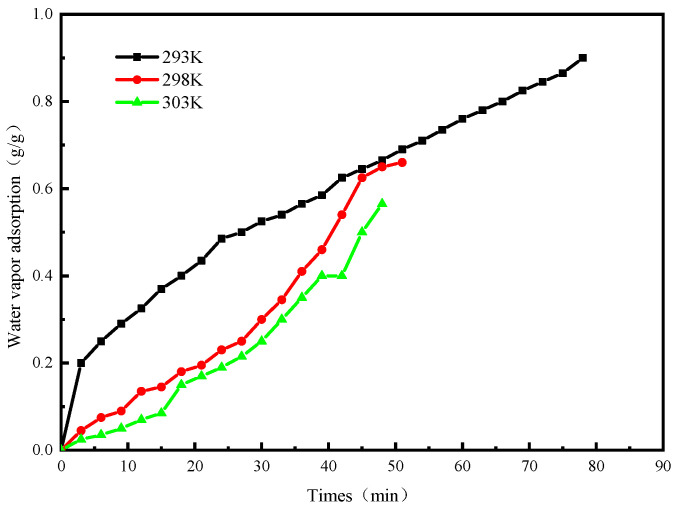
The curve of adsorption uptake of water vapor versus time.

Figure 13 shows the variation of system adsorption over time at different adsorption temperatures. It can be seen from the figure that when T_e_ = 288 K, T_c_ = 308 K, T_g2_ = 353 K, the adsorption temperature was 293 K, the adsorption capacity was 0.9 g/g; when the adsorption temperature was 298 K, the adsorption capacity was 0.66 g/g; when the adsorption temperature was 303 K, the adsorption capacity was 0.565 g/g. From this we can see that as the adsorption temperature decreases, the amount of water vapor adsorption in the system gradually increases. The increase in the adsorption temperature causes the relative pressure to decrease. It can be seen from the adsorption isotherm of Figure 5 that as the relative pressure decreases, the equilibrium adsorption capacity decreases.

**Figure 14:**
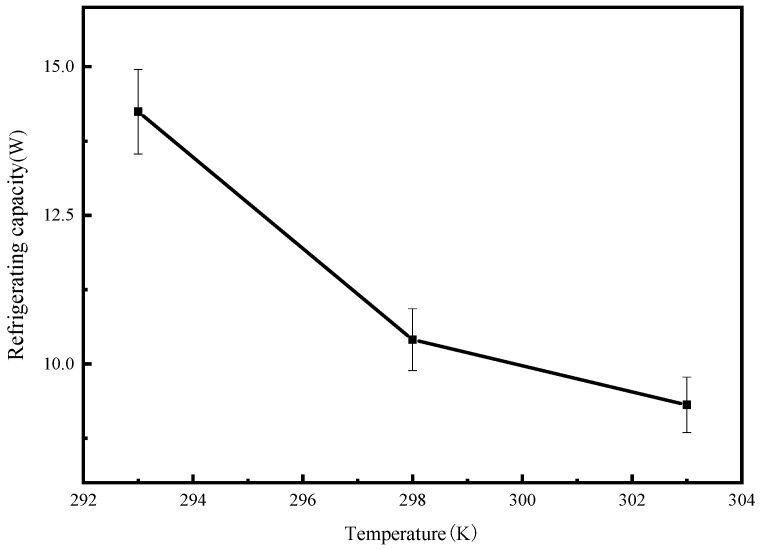
Cooling capacity of the system at different adsorption temperatures.

It can be seen from Figure 14 that the cooling capacity decreases with increasing adsorption temperature. When the adsorption temperature was 293 K, the system cooling capacity increased by 4.94W compared with that of when the adsorption temperature was 303 K.

**Figure 15:**
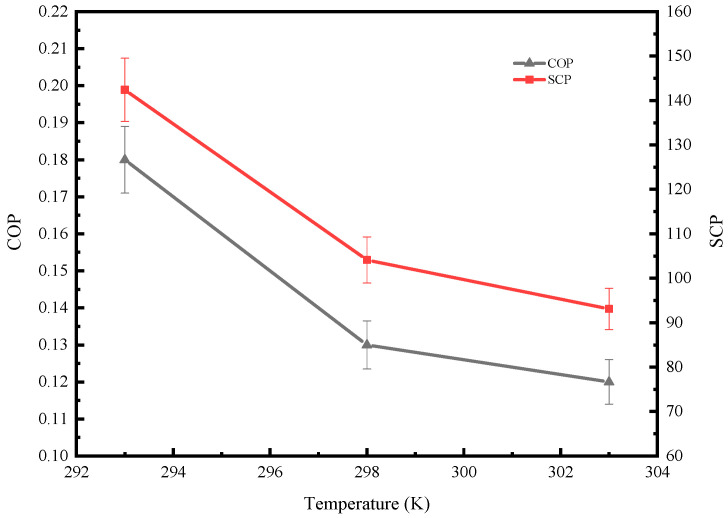
COP and SCP of the system at different adsorption temperatures.

It can be seen from Figure 15 that the COP and SCP of the system decrease with the increase in adsorption temperature, and the downward trend is very obvious. When the adsorption temperature was 293 K, the COP and SCP of the system were 0.18 and 142.4 W/kg, respectively. When the adsorption temperature was raised to 303 K, the COP and SCP of the system were reduced to 0.12 and 93 W/kg, respectively, and the performance of the system was nearly doubled compared with the adsorption temperature of 293 K. Therefore, in the adsorption refrigeration system, the adsorption temperature has a significant influence on the performance of the system.

### 2.7. Comparison of Experimental Results with Simulation Results

The experimental results and simulation results are summarized in Table 2 and Table 3. The comparison shows that the simulation results basically reflect the variation of system performance under different working conditions. However, as far as the specific values are concerned, the experimental results are slightly lower than the simulation results, mainly because of the following three reasons: (1) The power of the electric heating furnace of the adsorption bed was too large, and the heat generated during the desorption process was excessive, resulting in the system sensible heat increasing and the system performance decreasing. (2) The specific heat capacity selected during the simulation process was mostly fixed, and the specific heat capacity changed with the experimental temperature during the experiment; (3) The leakage heat of the whole experimental system was better. There was more, and the simulation process ignored the heat that the system loses into the environment.

## 3. Conclusions

1. MIL-101(Cr) was successfully synthesized and the structure of MIL-101(Cr) remained unchanged after CaCl_2_ was added.
2. With the increase in CaCl_2_ content, the total pore volume and pore diameter distribution of MIL-101(Cr)/CaCl_2_ composite material gradually decreased. When CaCl_2_ concentration reached 30%, the pore volume of MIL-101(Cr) was almost completely blocked by molecules.
3. The COP simulation model showed that the optimal desorption temperature and COP were 351 K and 0.24, 0.1 higher than MIL-101(Cr).

## 4. Materials and Methods

### 4.1. Reagents and Materials

Chromium nitrate nonahydrate (Cr(NO_3_)_3_·9H_2_O, ≥99% purity, Aladdin Biotechnology Co. Ltd., Shanghai, China), terephthalic acid (H_2_BDC, ≥99.0% purity), hydrofluoric acid (HF, ≥40% purity, Maclean Biotechnology Co. Ltd., Shanghai, China), *N*,*N*-Dimethyl formamide (DMF, ≥99.9% purity), ammonium fluoride (NH_4_F, ≥99.99% purity), ethanol (≥99.7% purity, Beijing Tongguang Fine Chemical Co. Ltd., Beijing, China), anhydrous calcium chloride (≥99.99% purity), and distilled water (prepared by the laboratory) were used in the experiments.

### 4.2. Synthesis of MIL-101(Cr)

The parent MIL-101(Cr) was synthesized by hydrothermal synthesis using the procedure reported by Yang [19,20].

### 4.3. Synthesis of MIL-101(Cr)/CaCl_2_ Composites

MIL-101(Cr)/CaCl_2_ composites were prepared by the immersion method [17]. About 0.4 g of MIL-101(Cr) was weighed and dried in an oven at 100 °C for 8 h. The MIL-101(Cr) sample was cooled to room temperature and then immersed in 10%, 20%, and 30% CaCl_2_ solutions (soaked at room temperature for 2 h). Finally, the soaked material was subjected to centrifugation, washed with deionized water, and centrifuged again to remove excess calcium chloride. The sample was collected and completely dried overnight in a vacuum oven at 373 K.

### 4.4. Materials Characterization

X-ray diffraction (XRD) was used to analyze the image of the composite. The diffraction peak was used to determine whether the composite was successfully synthesized. The thermogravimetric analysis (TG) was used to test the relationship between material quality and temperature, and its composition and thermal stability were studied. The specific surface area and pore structure of the composite were analyzed by N_2_ adsorption–desorption isotherm. The water vapor adsorption isotherm, adsorption kinetics and differential adsorption heat curves of the composite adsorbent were obtained by the gravimetric method, and the huge space and potential in the adsorption refrigeration application were preliminarily determined.

### 4.5. Measurement of Water Vapor Adsorption Isotherms

The water vapor adsorption isotherm, adsorption kinetics curve and adsorption heat curve of the material were tested by Conta’s DVS Advantage water vapor adsorption instrument. A weight about 40 mg of the sample was vacuum dried at 373 K to remove the solvent molecules from the material and adsorb the water vapor into the pores. The material was then cooled to room temperature and placed in the crucible of the instrument, then the interface was clicked on to run the program. During the test, the temperature of the adsorption bed and the crucible were set to 298 K and 308 K, respectively.

### 4.6. Experimental Rig of Single-Bed Adsorption System with New Type Adsorbent Filling Method

**Figure 16:**
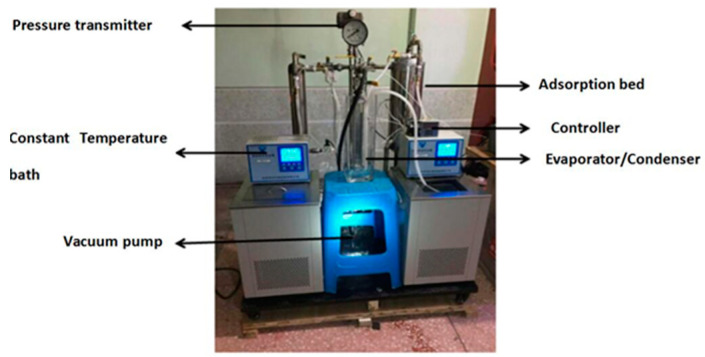
Adsorption refrigeration system performance test device.

**Figure 17:**
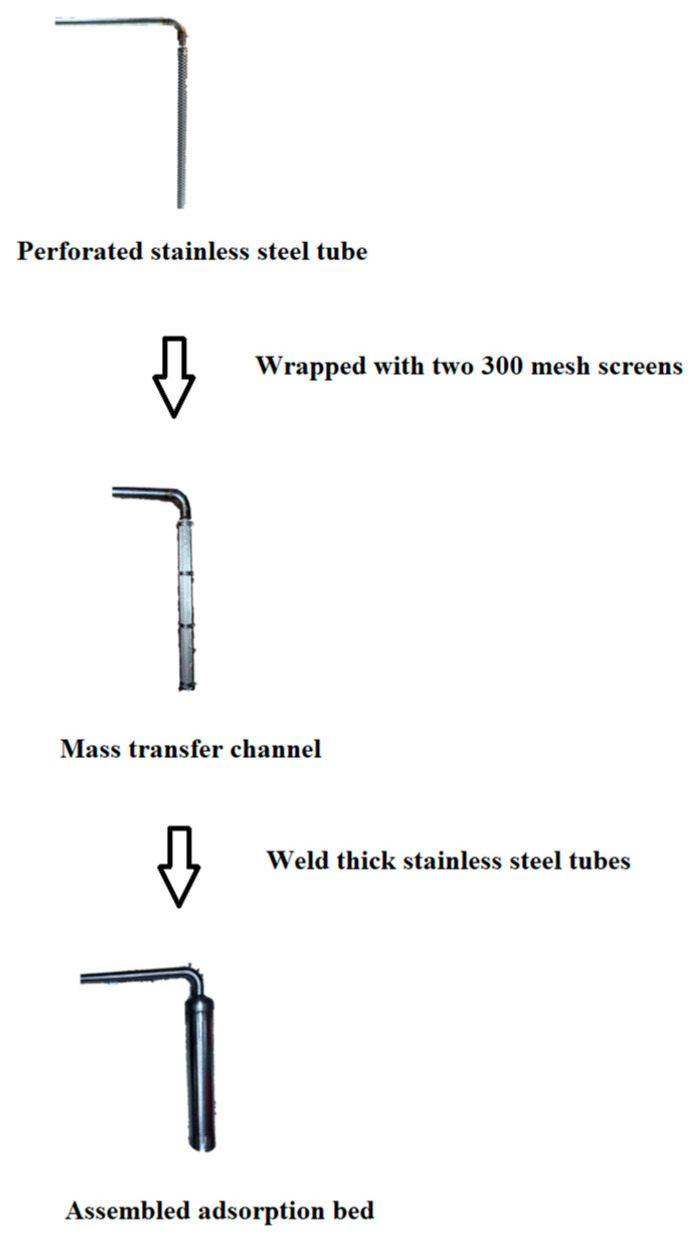
Production process of the adsorption bed.

**Figure 18:**
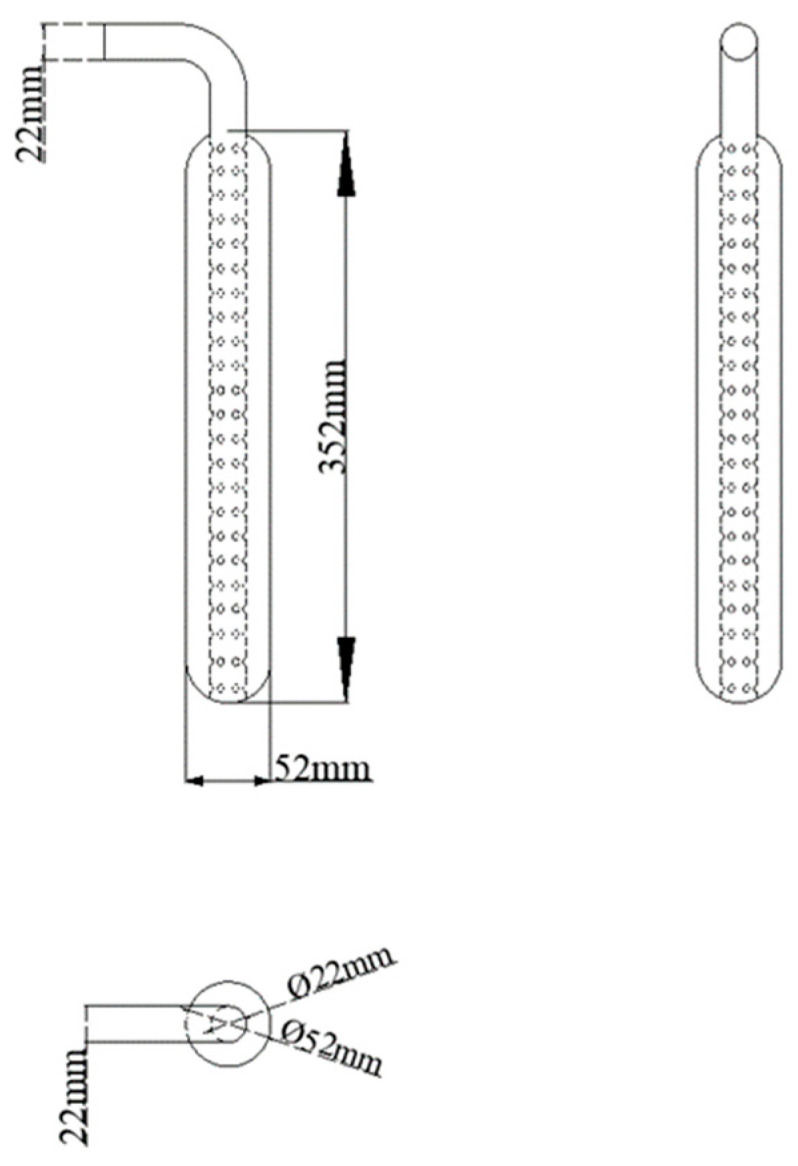
Adsorption bed structure.

The experimental platform of adsorption refrigeration system adopts the same structure as the previous study [21], as shown in Figure 16. We changed the adsorption bed filling method. Figure 17 shows the adsorption bed fabrication process. We took a 30 cm long stainless steel tube with a diameter of 22 mm, opened a certain number of 5 mm holes, this was the mass transfer hole. The empty stainless steel tube was wrapped with two 300-mesh screens, and clamped to the stainless steel tube with a clamp to prevent the adsorbent powder from being drawn into the pipeline of the system during vacuuming, which affects the normal operation of the experiment. We welded the stainless steel tube with the mass transfer holes into the center of a thick stainless steel tube (adsorption bed) with a length of 30 cm and a diameter of 52 mm. At this time, the inner diameter of the small stainless steel tube was the mass transfer channel of the whole adsorption bed. The composite adsorbent material was then filled into the gap between the thick stainless steel tubes, and the adsorbent bed was welded and sealed and attached to the system via a vacuum ball valve. The adsorption bed had sufficient mass transfer space to enhance the heat and mass transfer capacity of the adsorption bed. The structure of the adsorption bed is shown in Figure 18.

### 4.7. Experimental Procedure for Performance Test of Adsorption Refrigeration System

**Figure 19:**
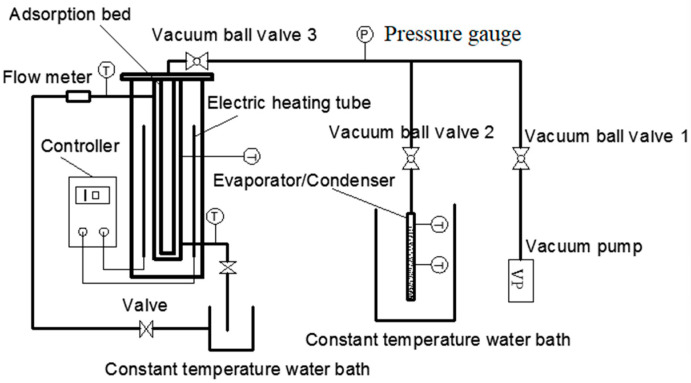
Schematic diagram of adsorption performance test.

The schematic diagram of the experimental system is shown in Figure 19. The experimental steps are as follows:

Adsorption bed heating vacuum process: because the system requires high vacuum, it is necessary to heat the adsorption bed before the cycle starts, set the heating temperature to 100 °C, then turn on the vacuum pump for 30 s. Then the vacuum ball valve 1 and the vacuum ball valve 3 are opened, and the adsorption bed is heated and evacuated until the internal pressure of the adsorption bed is maintained at about −101kPa. The vacuum ball valve 1 is closed, the constant temperature water bath is turned on, and the adsorption bed is cooled to the adsorption temperature.

To remove excess air from the evaporator/condenser: open the vacuum ball valve 2 on the basis of the previous step and remove excess air from the evaporator/condenser until the pressure in the evaporator/condenser reaches the evaporation temperature. Saturate the pressure, then turn off the vacuum ball valve 1 and then turn off the vacuum pump.

Adsorption–evaporation process: the vacuum ball valve 2 and the vacuum ball valve 3 are opened, and the adsorption bed is connected with the evaporator. The refrigerant in the evaporator changes from a liquid state to a gaseous state and enters the adsorption bed to be adsorbed by the adsorbent, and then the temperature and pressure in the adsorption bed will increase. When the valve is not opened, the pressure in the evaporator remains at the condensation pressure, and the pressure difference between the adsorber and the evaporator is the difference between the condensation pressure and the evaporation pressure. At the beginning of the adsorption process, the pressure inside the evaporator will gradually decrease to the evaporation pressure, and the pressure difference between the adsorber and the evaporator will gradually decrease to zero. At the same time, the water bath that opens the adsorbent bed cools the adsorbent bed to ensure that the adsorbent bed temperature is maintained at the adsorption temperature. The liquid water in the evaporator evaporates into a heat exchange between the water vapor and the external environment, so that the temperature of the evaporator water bath is continuously reduced as the adsorption process progresses, thereby generating a refrigeration effect. The adsorption process ends when the adsorbent bed pressure is the same as the evaporator pressure. The initial adsorption time, changes in evaporator/condenser level, and adsorption end time are recorded.

Heating process: the adsorption bed is heated before the desorption process. The adsorption bed is heated to 100 °C by an electric heating tube, during which the internal temperature and pressure of the adsorption bed are both increased. When the pressure in the adsorption bed is greater than the pressure in the condenser, the temperature rise is completed.

Desorption–condensation process: set the temperature of the water bath where the condenser is set to the condensation temperature and open the vacuum ball valve 2. Due to the pressure difference between the adsorption bed and the condenser, the gaseous refrigerant will enter the condensation through the pipeline under the action of pressure and with the desorption, the pressure difference between the adsorber and the condenser decreases. The condenser condenses into a liquid refrigerant, and the temperature of the condenser water bath increases. When the internal pressure of the condenser is equal to the internal pressure of the adsorbent bed, the condensation process ends.

Cooling process: when the desorption process is finished, the adsorption bed is still in a high temperature and high pressure state, which is not conducive to the next cycle, so the adsorption bed needs to be cooled before the start of the next cycle. The circulating water is opened to the adsorption bed. When the pressure in the adsorption bed drops to the saturation pressure corresponding to the evaporation temperature, the cooling process ends and the next adsorption cycle begins.

The parameters of electric heating tube, pressure transmitter and constant temperature water bath used in the above experiment are shown in Table 4, Table 5 and Table 6.

## 5. Mathematical Model of the Single-Bed Adsorption System

### 5.1. Basic Assumptions of the Theoretical Model

For an adsorption refrigeration system, its internal energy flow is a complex process, and it is impossible to fully express such a complicated energy flow process by mathematical expression. There is no need to consider such a complicated problem in the engineering application, so the complex problem can be simplified in mathematical modeling, regardless of the heat transfer process of internal load during energy flow, and the lumped parameter method is used to describe the energy flow process. The system parameters and constants involved in the simulation process are shown in Table 7.

In order to simplify the calculation, the following assumptions were made:

(1) The temperature and vapor pressure inside the entire adsorption bed are uniform.
(2) The water is uniformly adsorbed by the MIL-101(Cr)/CaCl_2_ composite material, and the water is liquid in the MIL-101(Cr)/CaCl_2_ composite material.
(3) The pressure difference between the adsorption bed and the condenser are ignored, the pressure difference between the adsorption bed and the evaporator are ignored.
(4) The heat conduction of the casing connected between the adsorption bed and the condenser or the evaporator is ignored, assuming complete insulation between the adsorption bed, the condenser and the evaporator.
(5) In addition to accidental heat exchange between hot water, cooling water and chilled water, the system ignores the cold/heat in the environment.
(6) The specific heat capacity of the MIL-101(Cr)/CaCl_2_ composite, the specific heat capacity of the adsorbent bed and the specific heat capacity of the refrigerant are constant.

### 5.2. Mathematical Model of the Basic Cycle

The simulation model of this cycle is based on COP Equation (1).

(1) $$\mathrm{COP}=\frac{Q_{ref}-Q_{eva}}{Q_{h}+Q_{g}}$$

The seven heat calculation formulas involved are as follows:

#### 5.2.1. The Sensible Heat Absorbed by the Adsorption Bed During Constant Volume Boost (*Q_h_*)

(2) $$Q_{h}=\int_{T_{a2}}^{T_{g1}}C_{a}M_{a}dT+\int_{T_{a2}}^{T_{g1}}C_{lc}M_{a}x_{a2}dT+\int_{T_{a2}}^{T_{g1}}C_{m}M_{m}dT$$

where *T_g_*_1_ is the initial analytical temperature (K), *T_a_*_2_ is the adsorption temperature (K), *C_a_* is the specific heat capacity of the adsorbent (kJ·kg^−1^·K^−1^), *C_lc_* is the specific heat capacity of the refrigerant (kJ·kg^−1^·K^−1^), *C_m_* is the specific heat of the metal in the adsorbent bed (kJ·kg^−1^·K^−1^), *M_a_* is the mass of the adsorbent (kg), *M_m_* is the mass of the adsorbent bed (kg), and *X_a_*_2_ is the adsorption amount of the adsorption bed at the end of adsorption of the adsorption line corresponding to the *T_a_*_2_ temperature point. In Equation (2), the first term represents the sensible heat of the adsorbent, the second term represents the sensible heat of the liquid refrigerant, and the third term represents the sensible heat of the adsorbent bed.

#### 5.2.2. Heat Absorbed during Desorption (*Q_g_*)

(3) $$Q_{g}=\int_{T_{g1}}^{T_{g2}}C_{a}M_{a}dT+\int_{T_{g1}}^{T_{g2}}C_{lc}M_{a}xdT+\int_{T_{g1}}^{T_{g2}}C_{m}M_{m}dT-\int_{T_{g1}}^{T_{g2}}M_{a}h_{d}dT$$

where *T_g_*_2_ is the desorption temperature (K), x is the adsorbent adsorption amount (kg·kg^−1^), and *h_d_* is the desorption heat (kJ·kg^−1^). In Equation (3), the first term represents the sensible heat of the cooled adsorbent, the second term represents the sensible heat of the refrigerant, and finally the sensible heat required to cool the adsorbent bed.

#### 5.2.3. The Sensible Heat Taken Away by the Cooled Adsorption Bed (*Q_c_*)

(4) $$Q_{c}=\int_{T_{a1}}^{T_{g2}}C_{a}M_{a}dT+\int_{T_{a1}}^{T_{g2}}C_{lc}M_{a}x_{d2}dT+\int_{T_{a1}}^{T_{g2}}C_{m}M_{m}dT$$

where *T_a_*_1_ is the initial adsorption temperature (K), and *x_d_*_2_ is the adsorption amount (kg·kg^−1^) of the adsorbent at the end of desorption corresponding to the *T_g_*_2_ temperature point. The first term in Equation (4) represents the sensible heat of the adsorbent, the second term represents the sensible heat of the refrigerant remaining in the adsorbent bed, and the third term represents the sensible heat of the adsorbent bed.

#### 5.2.4. Heat Taken Away by the Cold Source during Adsorption (*Q_ad_*)

(5) $$\begin{array}{ll} Q_{ad}=\int_{T_{a2}}^{T_{a1}}C_{a}M_{a}dT & +\int_{T_{a2}}^{T_{a1}}C_{lc}M_{a}xdT\\ & +\int_{T_{a2}}^{T_{a1}}C_{m}M_{m}dT-\int_{0}^{T_{a}-T_{e}}C_{pc}M_{a}\Delta xdT+\int_{T_{a2}}^{T_{a1}}M_{a}h_{a}dT \end{array}$$

where *C_pc_* is the constant pressure specific heat of the gaseous working fluid (kJ·kg^−1^·K^−1^), and ha is the heat of adsorption (kJ·kg^−1^). The last term in Equation (5) is the sensible heat of the vaporized working gas temperature rising from the evaporation temperature to *T_a_*.

#### 5.2.5. Refrigerating Capacity (*Q_ref_*)

(6) $$Q_{ref}=M_{a}L\Delta x$$

where *L* is the latent heat of vaporization of water(kJ·kg^−1^) is cyclic adsorption (kg·kg^−1^).

#### 5.2.6. Heat Released by Condensation (*Q_cond_*)

(7) $$Q_{cond}=M_{a}L\Delta x+\int_{T_{c}}^{T_{g2}}C_{pc}M_{a}\frac{d_{x}}{d_{t}}dT$$

where *T_c_* is the condensation temperature (K). In Equation (7), the first term is the latent heat of saturated vaporization, and the second term is the sensible heat released by refrigerant vapor during condensation.

#### 5.2.7. Sensible Heat Released by Liquid Refrigerant from *T_c_* to Evaporation Temperature *T_e_* (*Q_eva_*)

(8) $$Q_{eva}=\int_{T_{c}}^{T_{e}}C_{lc}M_{a}\Delta xdT$$

where *T_e_* is the evaporation temperature (K). In fact, as adsorption refrigeration is a complicated heat and mass transfer process, there are various heat losses, so it is very difficult to accurately calculate all kinds of heat. However, it can be theoretically useful to analyze the cycle through these equations.

#### 5.2.8. Expressions for *T*_*g*1_ and *T*_*a*1_

Before the beginning of the adsorption process, after opening the valve connecting the adsorber and the evaporator, the gas in the evaporator releases heat from the condensation temperature *T_c_* to the evaporation temperature *T_e_*, and the pressure drops from the condensation pressure *P_c_* to the evaporation pressure *P_e_*. The adsorption amount remains unchanged during the process. According to the ideal gas state equation, its relationship is shown in Equation (9). Before the desorption process begins, there is a preheating process in the adsorbent. The gas temperature in the adsorbent rises from *T_a_*_2_ to the initial desorption temperature *T_g_*_1_, while the pressure rises from the evaporation pressure *P_e_* to the condensing pressure *P_c_*, and the gas quantity remains unchanged. The equation is also shown in Equation (10). Similarly, the complete desorption temperature *T_g_*_2_ and the initial adsorption temperature *T_a_*_1_ satisfy the Equation (11). Equations (12) and (13) are obtained from simultaneous Equations (9)–(11).

(9) $$\frac{P_{c}}{P_{e}}=\frac{T_{c}}{T_{e}}$$

(10) $$\frac{P_{c}}{P_{e}}=\frac{T_{g1}}{T_{a2}}$$

(11) $$\frac{P_{c}}{P_{e}}=\frac{T_{g2}}{T_{a1}}$$

(12) $$T_{g1}=\frac{T_{c}T_{a2}}{T_{e}}$$

(13) $$T_{g2}=\frac{T_{c}T_{a1}}{T_{e}}$$

## Figures and Tables

**Table 1:** Pore structure parameters of MIL-101(Cr) and composite materials.

Sample	BET Specific Surface Area (m^2^/g)	Pore Diameter (cm^3^/g)
**MIL-101(Cr)**	2824	1.362
MIL-101(Cr)/CaCl_2_-10%	1977	0.745
MIL-101(Cr)/CaCl_2_-20%	1307	0.509
MIL-101(Cr)/CaCl_2_-30%	193	0.071

**Table 2:** Experimental results and simulation results at different evaporation temperatures.

Evaporating Temperature (K)		Q_ref_ (kJ)	COP
288	Simulation result	79.3	0.15
	Experimental result	73.95	0.103
293	Simulation result	121	0.192
	Experimental result	110.9	0.154
298	Simulation result	125.2	0.23
	Experimental result	123	0.172

**Table 3:** Experimental results and simulation results at different adsorption temperatures.

Adsorption Temperature (K)		Q_ref_ (kJ)	COP
293	Simulation result	142	0.21
	Experimental result	128.2	0.18
298	Simulation result	119	0.19
	Experimental result	93.67	0.13
303	Simulation result	100	0.17
	Experimental result	83.81	0.12

**Table 4:** The main technical parameters of the electric heating tube.

Content	Value
Temperature Range (K)	273–383
Accuracy (K)	0.1
Power Voltage (Hz/V)	50/220
Power (W)	200

**Table 5:** The main technical parameters of the pressure transmitter.

Content	Value
Power Supply (V)	24
Operating Temperature Range (℃)	−30–80
Signal (mA)	4–20
Range (kPa)	−100–1600
Accuracy (kPa)	0.1

**Table 6:** The main technical parameters of the constant temperature bath.

Content	Value
Temperature Range (K)	268–373
Temperature Fluctuation (K)	±0.05
Accuracy (K)	0.1
Volume (L)	7.5
Circulating pump flow (L/min)	6
Power Voltage (Hz/V)	50/220

**Table 7:** Physical property parameters used in the simulation.

Symbol	Term	Value	Unit
*M_a_*	Mass of the adsorbent	0.1	kg
*M_m_*	Mass of the adsorbent bed	2	kg
*C_lc_*	Specific heat capacity of the refrigerant	4.18	kJ/kg·K
*C_m_*	Specific heat of the stainless steel	0.5	kJ/kg·K
L	latent heat of vaporization of water	2465	kJ/kg

## Nomenclature

*T_g_*_1_	initial desorption temperature, K
*T_g_*_2_	desorption temperature, K
*C_a_* (T)	specific heat capacity of the adsorbent, kJ·kg^−1^·K^−1^
*M_a_*	adsorbent mass, kg
*T_a_*_1_	initial adsorption temperature, K
*T_a_*_2_	adsorption temperature, K
*C_lc_* (T)	specific heat capacity of water, kJ·kg^−1^·K^−1^
*C_m_* (T)	specific heat capacity of stainless steel, kJ·kg^−1^·K^−1^
*M_m_*	adsorption bed mass, kg
*Q_h_*	sensible heat absorbed by the adsorption bed during constant volume boost, kJ·kg^−1^
*Q_g_*	heat absorbed during the desorption process, kJ·kg^−1^
*h_d_*	desorption heat, kJ·kg^−1^·K^−1^
*h_a_*	heat of adsorption kJ·kg^−1^
*Q_ref_*	cooling capacity, kJ·kg^−1^
*L*	latent heat of vaporization of water, kJ·kg^−1^
Δ*x*	circulating adsorption capacity, kg·kg^−1^
*X_a_*_2_	water adsorption capacity in gas–solid-phase equilibrium at the time of adsorption in adsorption temperature, kg·kg^−1^
*X_d_*_2_	water adsorption capacity in gas–solid-phase equilibrium at the time of desorption in desorption temperature, kg·kg^−1^
*Q_eva_*	sensible heat of liquid refrigerant from Tc to evaporation temperature Te, kJ·kg^−1^
*T_c_*	condensation temperature, K
*T_e_*	evaporation temperature, K
